# Optical coherence tomography for identification of malignant pulmonary nodules based on random forest machine learning algorithm

**DOI:** 10.1371/journal.pone.0260600

**Published:** 2021-12-31

**Authors:** Ming Ding, Shi-yu Pan, Jing Huang, Cheng Yuan, Qiang Zhang, Xiao-li Zhu, Yan Cai

**Affiliations:** 1 Department of Respiratory Medicine, Southeast University Zhongda Hospital, Nanjing, Jiangsu, China; 2 School of Biological Sciences and Medical Engineering, Southeast University, Nanjing, Jiangsu, China; University of Oklahoma, UNITED STATES

## Abstract

**Objective:**

To explore the feasibility of using random forest (RF) machine learning algorithm in assessing normal and malignant peripheral pulmonary nodules based on *in vivo* endobronchial optical coherence tomography (EB-OCT).

**Methods:**

A total of 31 patients with pulmonary nodules were admitted to Department of Respiratory Medicine, Zhongda Hospital, Southeast University, and underwent chest CT, EB-OCT and biopsy. Attenuation coefficient and up to 56 different image features were extracted from A-line and B-scan of 1703 EB-OCT images. Attenuation coefficient and 29 image features with significant p-values were used to analyze the differences between normal and malignant samples. A RF classifier was trained using 70% images as training set, while 30% images were included in the testing set. The accuracy of the automated classification was validated by clinically proven pathological results.

**Results:**

Attenuation coefficient and 29 image features were found to present different properties with significant p-values between normal and malignant EB-OCT images. The RF algorithm successfully classified the malignant pulmonary nodules with sensitivity, specificity, and accuracy of 90.41%, 77.87% and 83.51% respectively.

**Conclusion:**

It is clinically practical to distinguish the nature of pulmonary nodules by integrating EB-OCT imaging with automated machine learning algorithm. Diagnosis of malignant pulmonary nodules by analyzing quantitative features from EB-OCT images could be a potentially powerful way for early detection of lung cancer.

## 1. Introduction

Pulmonary nodules are radiopaque densities seen in the lung parenchyma with a diameter of less than 3 cm^1^. Although possible causes of pulmonary nodules include many normal diseases, most of the early lung cancer patients are characterized by suffering from pulmonary nodules. Rapidly identifying the nature of pulmonary nodules could not only avoid unnecessary surgery, but also resect malignant lesions in a cost-effective manner. Nowadays, various imaging techniques have been employed for *in vivo* early detection of lung cancer, such as endobronchial ultrasound (EBUS) [1], computed tomography (CT) [2], PET/CT and PET/MR [3]. However, all these techniques whose resolution are millimeter-level are far away from ideal resolution for detecting the nature of pulmonary nodules.

As an optical imaging method, optical coherence tomography (OCT) is gaining credibility as a thoracic imaging tool in clinically [4, 5]. Using near-infrared light, cross-sectional microscopic images are created through optical interferometry, by detecting the backscattering of light as it interacts with tissue structures. Due to the use of low coherence light, OCT can produce images with resolution in the range of 5 to 15 μm, which allows it to visualize the different airway wall layers including mucosa, submucosa and cartilage [6–8]. Moreover, the penetration depth of OCT in the tissue (1–2 mm) is helpful to distinguish the invasive carcinoma from normal bronchial epithelium according to the changes of cellular and extracellular morphologies beneath the tissue surface. In the recent past, several pilot studies have been performed for lung cancer detection using OCT both *in vivo* and ex vivo [5–7, 9–11]. Studies by incorporating fluorescent bronchoscopy and endobronchial OCT (EB-OCT) on the abnormal airways demonstrated that EB-OCT could accurately capture the microscopic morphological changes of the cancerous mucosa [9]. The feasibility of EB-OCT to quantify separate airway wall layers and the satisfied correlation with histology and other imaging data have facilitated its application in the assessment of malignant pulmonary nodules [8].

To improve the accuracy of the early diagnosis of lung cancer, it is important to integrate the computer-aided diagnosis (CAD) into the processes of imaging pattern recognition and pulmonary nodules classification. In addition, automated image analysis methods can provide a robust diagnosis independent of visual interpretation limitations. Several pioneering work have explored the availability of using machine learning and deep learning algorithms to analyze the OCT images of breast cancer tissue [12, 13], human ovarian tissue [14] and atherosclerotic plaques [15]. Random Forest (RF) [16] model is an ensemble learning classifier that tries to achieve an accurate classification results by combining a large number of weak classifiers. RF combines multiple binary tree predictors where each tree is constituted by subset of features of a training-set which are randomly sampled and votes for a single class. The result of RF model classification is determined by the vote number of tree predictors. In recent years, the RF algorithm has been broadly utilized in the classification of medical images of human brain and prostate [17], breast cancer [18] and retina abnormalities [19]. However, computer-aided classification of pulmonary nodules based on EB-OCT image features has never been done before.

In this context, the main objective of this study is to explore the feasibility of using automated image analysis system to classify pulmonary nodules in malignant and normal based on EB-OCT images. To evaluate if significant differences exist between normal and malignant EB-OCT images regarding to the quantitative image features, we investigated attenuation coefficient and up to 56 different image features extracted from A-line and B-scan of EB-OCT images. These features were used as predictors for automatic identification of pulmonary nodules with potential malignancy using random forest (RF) machine learning algorithm. The sensitivity, specificity as well as the area under the receiver operating characteristic (ROC) curve were evaluated for diagnostic accuracy. The present analysis will be utilized for rapid, *in vivo* assessment of pulmonary nodules and help the surgeon/pathologist in early diagnosis, risk stratification, and prognosis of lung cancer patients.

## 2. Methods and materials

### 2.1 Patients

This study group comprised 31 patients with solitary pulmonary nodule (SPN) who underwent EB-OCT and corresponding pathological examinations at the Department of Respiratory Medicine, Zhongda Hospital, Southeast University between January 1^st^, 2018 and December 31^st^, 2020. Patient demographics were summarized in **Table 1**. This project was approved by the hospital ethics committee (Southeast University Zhongda Hospital, Ethical number: 2017ZDSYLL086-P01). All research was performed in accordance with the regulations of Southeast University Zhongda Hospital, and all patients provided written informed consent.

**Table 1:** Demographics of the patients.

No.	Gender	Age	Height (cm)	Weight (kg)	Years of smoking	Site of lesion	Size (cm)	CT features	Methods for diagnosis	Pathological results
1.	Male	64	168	58		RB6	1.7	SPN	TBLB	Inflammation
2.	Male	53	170	68	30	RB9	1.2	SPN (GGO)	TBLB+VATS	Adenocarcinoma
3.	Male	67	170	66	40	LB8	3.0	Mass (Solitary)	TBLB	Squamous cell carcinoma
4.	Male	64	171	64	20	RB5	3.0	SPN (GGO)	TBLB+VATS	Adenocarcinoma
5.	Female	57	159	60		RB2	2.9	Mass (Solitary)	TBLB+VATS	Adenocarcinoma
6.	Female	65	156	56		RB3	3.7	Mass (Solitary)	TBLB	Organizing pneumonia
7.	Female	71	155	65		LB3	4.1	Mass (Solitary)	TBLB	Organizing pneumonia
8.	Male	81	174	76	30	RB7	3.7	Mass (Solitary)	TBLB+CT guided percutaneous biopsy	Organizing pneumonia
9.	Male	76	170	61		RB3	5.3	Mass (Solitary)	TBLB	Inflammation
10.	Male	58	172	62	30	RB3	3.0	SPN	TBLB+VATS	Organizing pneumonia
11.	Male	62	171	71.5		LB9	5.1	Mass (Solitary)	TBLB	Organizing pneumonia
12.	Female	53	155	53		RB5	1.6	SPN (GGO)	TBLB	Adenocarcinoma
13.	Male	73	176	71	40	LB3	3.0	Mass (Solitary)	TBLB	Inflammation
14.	Female	58	163	56.5		RB6	1.2	SPN (GGO)	TBLB+VATS	Adenocarcinoma
15.	Male	77	174.5	63		RB7	2.8	SPN	TBLB+VATS	Adenocarcinoma
16.	Male	50	172	67	30	LB1	1.8	SPN (GGO)	TBLB	Inflammation
17.	Male	71	169	61.5	20	RB1	2.9	Mass (Solitary)	TBLB	Adenocarcinoma
18.	Male	62	171	55	30	RB1	2.4	SPN	TBLB+VATS	Organizing pneumonia
19.	Female	69	156	62		RB3	2.9	SPN	TBLB	Adenocarcinoma
20.	Female	78	155	47.5		LB1	2.0	SPN	TBLB	Inflammation
21.	Male	53	169	67.5		RB3	2.0	SPN	TBLB	Inflammation
22.	Male	65	177	59.5	30	LB1	2.2	SPN	TBLB	Inflammation
23.	Male	72	171	70	30	LB1	1.5	SPN	TBLB	Small cell lung cancer
24.	Female	79	156	55		LB1	2.3	SPN	TBLB	Adenocarcinoma
25.	Female	65	158	64		LB1	2.3	SPN	TBLB	Adenocarcinoma
26.	Female	85	155	43		RB3	2.7	SPN	TBLB	Adenocarcinoma
27.	Female	65	162	62		RB3	2.2	SPN	TBLB+VATS	Adenocarcinoma
28.	Male	34	171	60		RB9	2.6	SPN	TBLB	Inflammation
29.	Male	65	165	52.5		LB1	3.0	SPN	TBLB	Inflammation
30.	Male	37	173	65		RB4	1.8	SPN	TBLB	Inflammation
31.	Male	54	165	70	30	LB1	1.6	SPN	TBLB+CT guided percutaneous biopsy	Adenocarcinoma

**SPN**: Solitary pulmonary nodule.

**TBLB**: Transbronchial lung biopsy.

**VATS**: Video-assisted thoracic surgery.

**GGO**: Ground glass opacity pulmonary nodule.

Right upper lobe: Apical segment: **RB1**; Posterior segment: **RB2**; Anterior segment: **RB3**.

Right middle lobe: Lateral segment: **RB4**; Medial segment: **RB5**.

Right lower lobe: Superior segment: **RB6**; Medial basal segment: **RB7**; Anterior basal segment: **RB8**; Lateral basal segment: **RB9**; Posterior basal segment: **RB10**.

Left upper lobe: Apical segment: **LB1**; Posterior segment: **LB2**; Anterior segment: **LB3**; Superior lingula segment: **LB4**; Inferior lingula segment: **LB5**.

Left lower lobe: Superior segment: **LB6**; Medial basal segment: **LB7**; Anterior basal segment: **LB8**; Lateral basal segment: **LB9**; Posterior basal segment: **LB10**.

### 2.2 Data acquisition

All patients received chest CT screening and chest CT thin-layer reconstruction to obtain the 0.65 mm DICOM format CT data. The data were then imported into the navigation bronchoscope system (DirectPath, Olympus, Japan) to construct a three-dimensional bronchial tree, so as to locate and mark nodular lesions. Under the guidance of navigation, the bronchoscope (Olympus, Japan, outer diameter: 4.0 mm) was simultaneously sent to the marked target site. After that, the radial endobronchial ultrasound (R-EBUS, Olympus ME2 Plus, probe outer diameter: 1.4 mm) was inserted to scan the airway and the quantitative information were recorded such as the lesion range and the depth from the lesion to the target bronchial opening. According to the planned pathway generated by the navigation system, all 31 patients underwent examination by an OCT system (Guangdong Winstar Medical Technology CO., China and Tomophase Inc., Cambridge, MA, USA) which consists of an imaging computing system and a sterile detachable probe. The OCT probe is a catheter with a diameter of 1.7 mm and a length of 15 cm, sealed with a transparent outer sheath and a 1 mm long window near the tip for scanning and capturing images. The optical fiber is illuminated by a broadband light source operating at 1300 nm with 50 kHz sweeping rate. In both grayscale and color modes, the image acquisition speed is 20 frames per second, with an axial resolution of 15 μm axial, a lateral resolution of 25 μm, and a depth resolution of up to 3 mm. To obtain OCT images of airways tissues, the EB-OCT catheter was inserted to the target lesion or tissues around the lesion and then was fixed there under the real-time synchronous guidance. According to the lesion depth marked by the ultrasound, the EB-OCT probe was inserted to the pre-determined target site and then pulled backwards. The image data of both the lesion and surrounding tracheas were obtained and recorded during the scanning (see video in **S1 and S2 Vides**). Lung biopsy was pe2rformed after bronchoscope for all patients. Rapid-on-site cytology evaluation (ROSE) was used to determine the quality of the materials. The pathological results of biopsy specimens were obtained by immunohistochemistry analysis based on HE staining.

### 2.3 Follow-ups

For patients whose initial negative biopsy results showed high risk of lung cancer, CT-guided lung puncture and surgery were performed to obtain clinically proven pathological diagnosis. For patients suggested inflammation but without puncture or surgery, a follow-up of every 3 months was conducted till the lesion was completely absorbed and confirmed to be normal.

### 2.4 EB-OCT imaging procedure

#### 2.4.1 Imaging protocol

**Fig 1:**
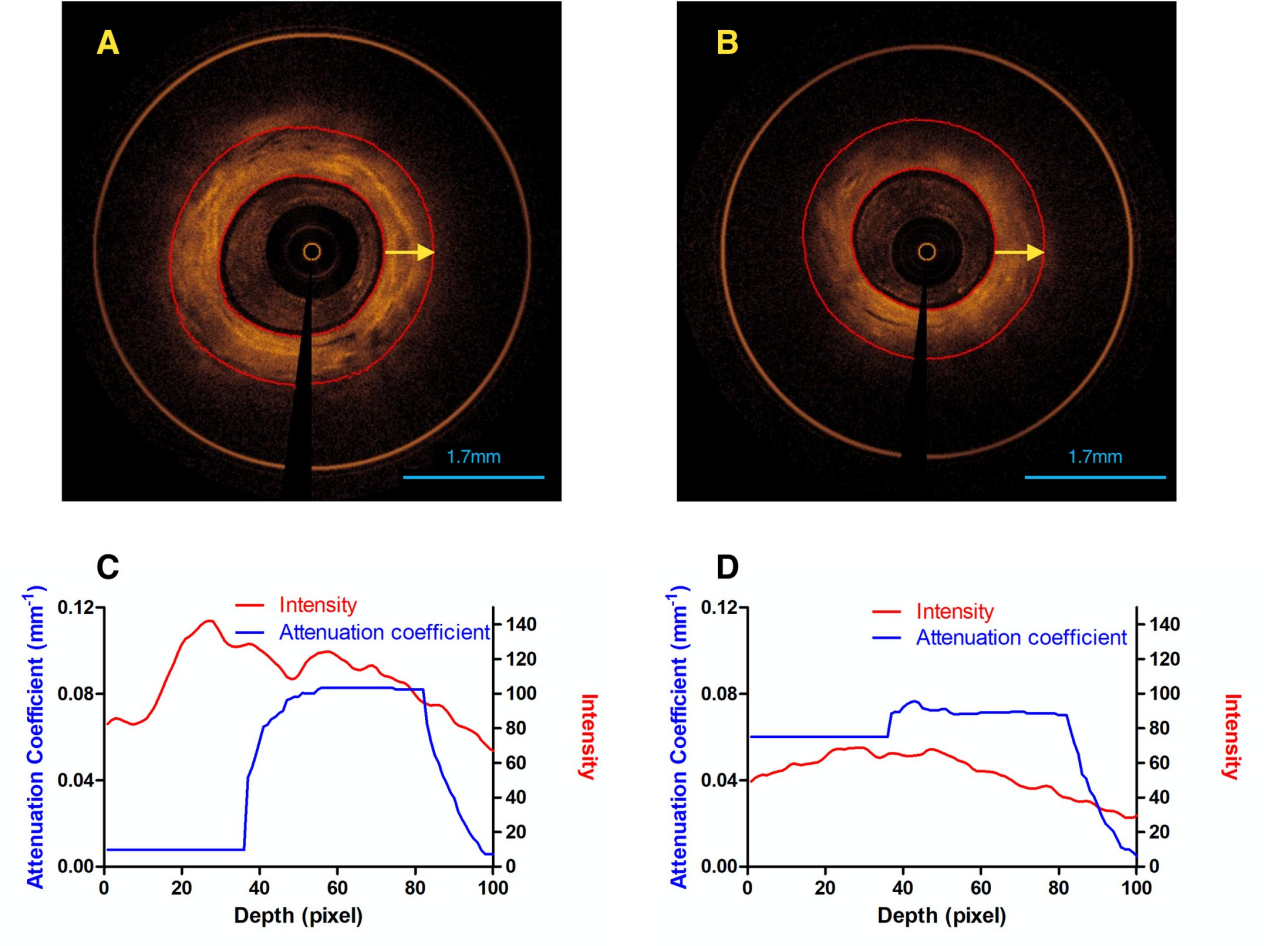
Normal and malignant airway tissue. EB-OCT images of (A) normal and (B) malignant airway tissue. ROI started from lumen boundary and ended at a depth of 100 pixels along A-line, corresponding to the region between two red circles. A-line was indicated by the yellow arrow. The average value of attenuation coefficient and intensity for each depth of 100 randomly selected A-lines were extracted from EB-OCT images of normal (C) and malignant (D) lesions, respectively.

To differentiate between normal and malignant tissue, several features were extracted from the acquired EB-OCT images. All the features were extracted from region of interest (ROI) with high signal-to-noise ratio (SNR). The guide wire and catheter artifacts were removed, and the lumen boundary was automatically recognized using the following procedures: (a) image transformation from polar coordinates to Cartesian coordinates; (b) spekle noise reduction by Gaussion filtering; (c) image binarization by using the Otsu’s method; (d) morphological operation. From the segmented lumen boundary, the tissue was selected at a depth of 1mm, corresponding to about 100 pixels (**Fig 1**).

The extracted features can be subdivided into two categories: (1) A-line-derived optical properties and (2) B-scan-derived image features. The detailed illustrations of these quantitative features were provided in the following sections. All data analysis was performed on MATLAB.

#### 2.4.2 Image features extraction

*A*. *Optical properties*. Attenuation coefficient was extracted from the A-lines. The attenuation coefficient ***μ_r_*** is a tissue property [20, 21] that can be measured independently in homogeneous media according to Lambert-Beer exponential decay curve [22, 23]:

$$I(z)=I_{0}T(z)S(z)exp\;(-{2\mu }_{r}z)$$ (1)

where T(z) and S(z) are point spread function and signal roll-off function, respectively. In time-domain OCT, S(z) is set to be one [24]. In this study, T(z) is simplified to be constant value (equal to one) [22, 23]. *I*_0_ is the locally intensity which is equal to the source intensity, and z is the penetration depth.

As biological tissues in general, the healthy airway tissue is composed by multiple layers (e.g. mucosal and submucosal layer, smooth muscle layer, cartilage). Previous studies demonstrated that attenuation coefficient *μ_r_* can be fitted to different tissue layers through individual fitting. To automatically fit the proposed model in different layers, the attenuation coefficient *μ_r_* of each depth was least-squares fitted using an iterative linear optimization model for each pixel along each A-line [25]. With a linear optimization, there was a unique optimum for each set of data, and results were independent of the initial guess required for an iterative nonlinear model. The cost function δ which was defined as the root of mean square difference between the measured OCT trace *I*(*z*) and the model fitted value was computed at every step k. Starting from the lumen boundary, the fitting window was extended until a decrease in fit quality was detected that δ in step k was bigger than the δ value in step k-1. Moving the window forward and searching for the longest window maximized the accuracy of the fitted attenuation coefficient, and the optimum values with smallest δ were stored. This procedure was repeated until the window encountered the end of the A-line.

To simplify the calculation, we randomly sampled 100 out of 5000 A-lines in each EB-OCT image and then took the average of *μ_r_* at the same depth (**Fig 1**). For each chosen A-line, we selected 100 pixels from the lumen boundary to exclude pixels with low SNR as much as possible.

*B*. *Image features*. Compared to normal airway tissues, malignant lesions are high scattering without clear layer structure [20], which can be measured by image features. A total of 56 features were extracted from B-scan images, which can be categorized into statistical feature and textural feature:

1. Statistical feature [26] (SF): (a) mean, (b) median value, (c) standard deviation, (d) skewness, (e) kurtosis.
2. Textural feature: (a) Fractal dimension analysis [27–29] (FDA): The Hurst coefficients for dimensions 1,2,3 and 4 were computed. (b) Fourier power spectrum [30–32] (FPS): radial sum and angular sum were calculated. (c) Spatial gray level dependence matrices [33, 34] (SGLDM) proposed by Haralick: angular second moment, contrast, correlation, sum of squares, inverse difference moment, sum average, sum variance, sum entropy, entropy, difference variance, difference entropy and information measures of correlation. Four values were computed for angles θ = 0, 45, 90 and 135 degrees, and for a chosen distance d = 1. The mean and range of the values over the four angles represents the features above. (d) Gray level difference statistics [34] (GLDS): contrast, energy, entropy and mean. Features above were calculated for displacements (Δ*x*, Δ*y*) = (0,1), (1,1), (1,0),(1,−1), and their mean values were taken. (e) Neighborhood gray tone difference matrix [35] (NGTDM): coarseness, contrast, busyness, complexity and strength. (f) Statistical feature matrix (SFM): coarseness, contrast, periodicity and roughness. (g) Laws’ texture energy measures [29, 36–38] (LTEM): LL-texture energy from LL kernel, EE-texture energy from EE-kernel, SS-texture energy from SS-kernel, LE-average texture energy from LE and EL kernels, ES-average texture energy from ES and SE kernels, and LS-average texture energy from LS and SL kernels. Detailed definition and formulas of these image features are provided in **S1 Text**.

### 2.5 Statistical analysis

In this study, the statistical software was SPSS 20.0 (SPSS Inc., Chicago, IL, USA), and the mapping software was Graphpad Prism 5.0 (Graphpad Inc., San Diego, USA). Population data and the percentages obtained were expressed in the form of Mean ± SD. The student’s t-test was used to investigate the differences between normal and malignant pulmonary nodules regarding to the attenuation coefficient and image features. A significance level of 0.05 was considered to be statistically significant.

### 2.6 Image classification

All aforementioned features with significant p-values were used as predict variables in the classifier and RF model classified it into two classes: normal and malignant tissue. In this study, the sample of EB-OCT images was firstly split based on subjects into two nonoverlapping subsets. In order to assess the robustness of the model, 10-fold cross-validation was performed to generate different training sets (70% images) and testing sets (30% images). The classification accuracy was calculated by comparing the clinically proven pathological results with the diagnostic results provided by automated classifier. The average classification accuracy, sensitivity and specificity were calculated.

## 3. Results

### 3.1 General patient characteristics

The 31 patients selected in this study had SPN lesions ranging from 1.2cm to 5.1cm, among which 15 cases were malignant tumors (13 cases of adenocarcinomas; 1 case of squamous cell carcinoma; 1 case of small cell lung cancer), 7 cases were inflammation, and the other 6 cases suggested organizing pneumonia. Among all the patients, 9 cases were diagnosed by surgery and 2 cases were diagnosed by CT guided percutaneous lung puncture. According to the pathological results of all 31 cases, 16 cases were demonstrated to be normal, whose lesions all disappeared during chest CT follow-ups. 6 cases were organizing pneumonia, among which 2 cases were diagnosed by surgery and 1 case was diagnosed by percutaneous puncture (**Table 1**).

### 3.2 Image features

Quantitative analysis of 1703 images of 31 patients were carried out to obtain the image features. **Table 2** presented the measured values (mean ± SD) and p-values of both optical properties and image features with significant differences between normal and malignant lesions.

**Table 2:** Attenuation coefficient and image features of normal and malignant airway tissues with significant p-values.

Features			Normal	Malignant	P Value
**Optical Properties**	Attenuation Coefficient		0.055±0.018	0.050±0.019	P = 0.044
**Image Features**	Statistical Features	Mean	19.015±2.907	16.883±1.444	P = 0.022
		Variance	7.347±3.301	4.683±2.347	P = 0.024
		Standard Deviation	27.741±2.650	25.044±1.113	P = 0.002
		Kurtosis	7.667±1.224	6.530±0.932	P = 0.012
	Fractal Dimension Texture Analysis	H2	0.268±0.021	0.255±0.010	P = 0.046
	Fourier Power Spectrum	Radial Sum	22329.124±2636.736	20062.536±1094.022	P = 0.007
		Angular Sum	7459.190±782.468	6654.144±751.668	P = 0.014
	Spatial Gray Level Difference matrices	Mean of Angular Second Moment	0.104±0.024	0.130±0.025	P = 0.012
		Range of Angular Second Moment	0.008±0.002	0.010±0.002	P = 0.031
		Mean of Contrast	79.518±10.863	70.401±5.743	P = 0.011
		Range of Contrast	29.222±4.691	25.297±2.306	P = 0.010
		Mean of Sum of Squares	788.191±117.119	642.880±37.404	P<0.001
		Range of Sum of Squares	0.644±0.061	0.545±0.065	P<0.001
		Mean of Inverse Difference Moment	0.420±0.039	0.452±0.036	P = 0.037
		Mean of Sum Average	40.072±5.902	35.795±2.940	P = 0.023
		Range of Sum Average	0.052±0.008	0.046±0.004	P = 0.021
		Mean of Sum Variance	3073.25±459.95	2501.12±148.29	P<0.001
		Range of Sum Variance	26.649±4.642	23.116±2.357	P = 0.018
		Range of Entropy	0.128±0.013	0.120±0.008	P = 0.049
		Mean of Difference Variance	50.765±5.608	46.476±3.189	P = 0.021
		Range of Difference Variance	18.408±2.571	16.476±1.521	P = 0.024
	Gray Level Difference matrices	Contrast	79.509±10.862	70.392±5.742	P = 0.011
		Energy	0.163±0.023	0.187±0.023	P = 0.017
		Mean	5.273±0.633	4.809±0.387	P = 0.029
	Neighborhood Gray Tone Difference Matrix	Contrast	0.490±0.101	0.401±0.054	P = 0.008
		Complexity	42023.943±4392.033	36220.977±2731.785	P<0.001
	Statistical Feature Matrix	coarseness	12.196±1.288	13.486±1.116	P = 0.011
	Laws Texture Energy Measures	LL	109129.317±10433.990	98313.561±4480.171	P = 0.002
		LE	6658.034±820.816	5960.603±406.741	P = 0.009

As shown in **Table 2**, the attenuation coefficient of normal tissue is found to be higher than malignant one (0.055±0.018 vs. 0.050±0.019, p = 0.044), which could be partly due to the presence of the extracellular matrix in the submucosal layer in normal nodules (**Fig 1)**. Besides, 29 image features showed significant differences between normal and malignant lesions. It is noteworthy that the malignant tissue image had lower standard deviation compared to normal tissue (25.044±1.113 vs. 27.741±2.650, p = 0.002), which may be attributed to the loss of layer structure and glandular tissues in malignant pulmonary nodules. Similarly, other image features in malignant lesions, such as skewness and kurtosis, entropy and features in the fractal dimension texture analysis, were found lower than normal one. These results showed consistency with histological evidence of the lack of layered structure in malignant airways.

### 3.3 Classification results

**Fig 2:**
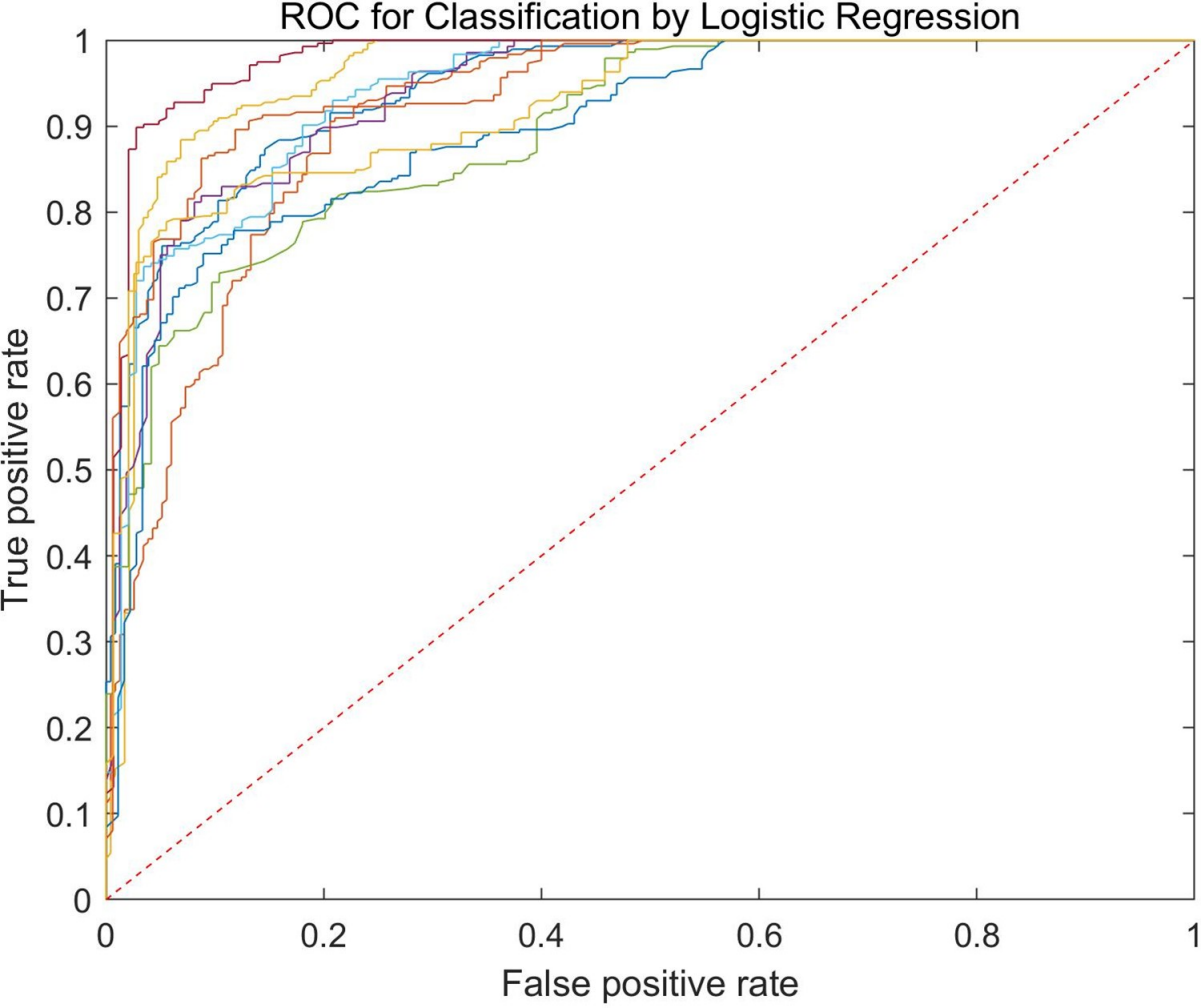
The ROC curve of the testing set of the classifier. Classification accuracy reached up to 81.4%, and sensitivity and specificity were 76.4% and 84.8% respectively.

The RF classifier was used for classification of normal and malignant airway tissues. A total of 1703 images were used in this experiment, out of which 70% were used for training while 30% were used for testing. **Fig 2** showed the ROC curves for testing sets. The average classification accuracy was up to 83.5%, and the average sensitivity and specificity were found to be 90.4% and 77.9% respectively.

## 4. Discussion

To the best of our knowledge, this is the first study to show the feasibility of automated identification of malignant pulmonary nodules in EB-OCT images via machine learning algorithm. Besides, this is the first report evaluating the quantitative image features of EB-OCT scans and investigating their significant differences between normal and malignant lesions. Importantly, the quantitative analysis from *in vivo* EB-OCT images was associated with clinically proven histopathological diagnosis. The prediction of automated classification was validated by the follow-up examinations of all patients.

**Fig 3:**
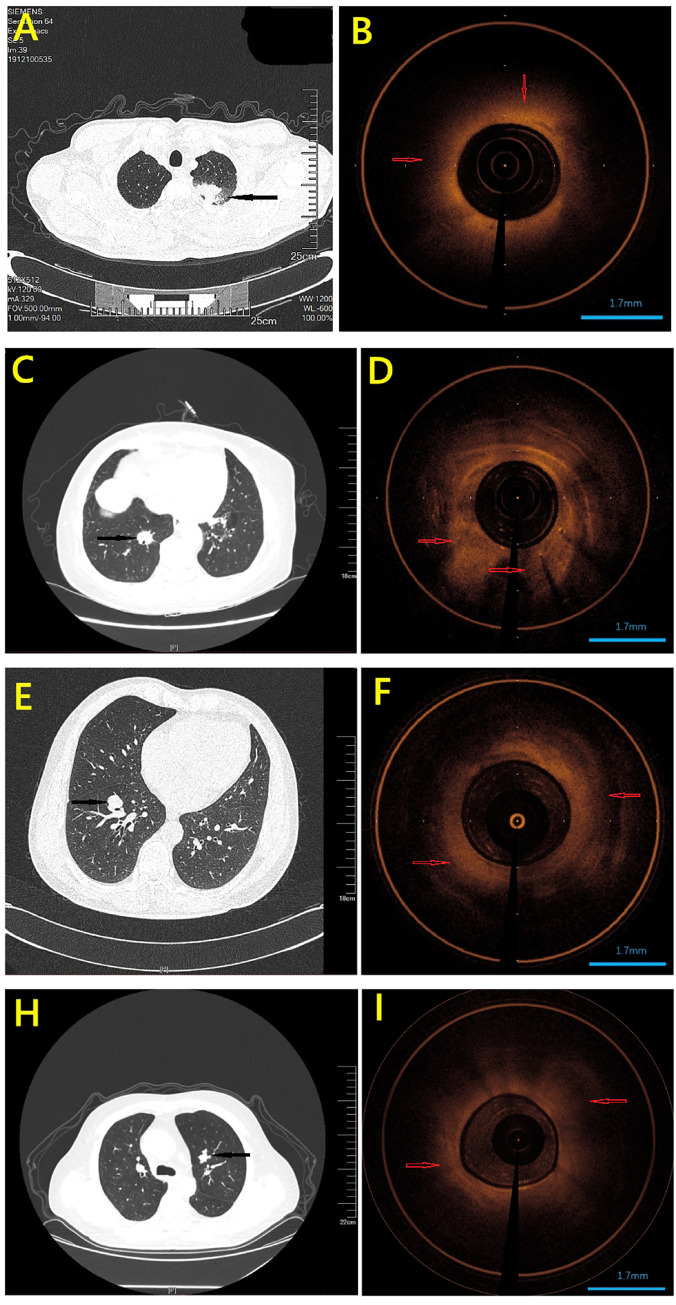
Pulmonary nodules of different cases on CT scan and corresponding OCT images. Although final pathology confirmed that they were different type lesions, including pneumonia (A&B), adenocarcinoma (C&D), squamous cell carcinoma (E&F) and small cell lung cancer (H&I), they have similar manifestations on CT scan (Black arrows point to lesions). OCT images demonstrated that normal lesion appeared homogeneous and had clear structure (B). In OCT images of malignant lesions (D F I), the lesions appear as unevenly distributed areas of high backscatter, resulting in the loss of layer structure and glandular tissue. Red arrows indicate the lesion areas.

Every year, millions of patients have an incidental pulmonary nodule identified on chest CT imaging, and the number of patients will only increase with implementation of lung cancer screening [39, 40]. However, the vast majority of patients cannot benefit from the detection of a pulmonary nodule since most of nodules are ultimately determined to be false-positive findings for lung cancer [41]. Therefore, it is important to develop strategies to determine if a small nodule is malignant when first identified. However, the traditional imaging test, such as CT and R-EBUS, cannot provide sufficient information to detect malignant lesions accurately [42–44]. For example, the CT images of 5 cases of patients in this study showed ground glass nodules (GGO). Unlike solid lung nodules, even if the probe of EBUS was performed inside the GGO nodules accurately, only the "blizzard-like" ultrasound images could be observed [45]. The features of these kind of images were too similar to that of inflammation and normal lung tissues to be identified [46]. Another example, **Fig 3** showed pulmonary nodules of different cases on CT scan and corresponding OCT images (an OCT image for healthy trachea was provided in **S1 Fig**). Although final pathology confirmed that they were different type lesions, including pneumonia (A&B), adenocarcinoma (C&D), squamous cell carcinoma (E&F) and small cell lung cancer (H&I), they have similar manifestations on CT scan. To overcome these issues, an alternative imaging technique is needed which will perform the real-time, noninvasive and rapid screening with high resolution.

Near infrared-based OCT is a novel imaging technique that combined with bronchoscopy generates highly detailed images of the airway wall [8]. The feasibility of EB-OCT to identify the human airway wall areas in total and in sublayers has been demonstrated in several pilot work [4, 47]. In addition, the comparison of ex vivo and *in vivo* OCT images with histology in human airways has been investigated [6, 7, 48]. However, the quantitative analysis of image features derived from airway OCT scans and the correlation with specific lesion are remain unknown. In this study, we investigated attenuation coefficient and other 56 image features extracted from A-line and B-scan OCT images. The p-value obtained from the student’s t-test suggested that there are significant differences of attenuation coefficient and 29 image features between normal and malignant tissues. Further analysis was performed to evaluate the association between the image features and histopathological subtypes (such as inflammatory) of the lesions (**Table 3**). One-way ANOVA test was carried out to examine if significant differences exist among EB-OCT images on three groups (normal, malignant and inflammatory). It was found that 7 features, including SF kurtosis, GLDS variance and NGTDM entropy, were significantly different (p<0.05) in these three groups. Remarkably, there was no significant difference in the optical property, i.e., the attenuation coefficient, among the three groups, which suggested that more specific image features should be addressed in the quantitative analysis of EB-OCT images of inflammatory patients.

**Table 3:** Image features of normal, malignant and inflammatory airway tissues with significant p-values.

	Features		Normal	Malignant	Inflammatory	P-Value
**Image Features**	Statistical Features	Standard deviation	28.36±2.03	25.52±0.61	25.94±0.84	P = 0.048
		Skewness	2.22±0.21	2.0±0.18	1.93±0.11	P = 0.029
		Kurtosis	8.02±1.19	6.42±0.82	6.28±0.5	P = 0.007
	Fourier Power Spectrum	Angular Sum	7840.45±606.53	6737.52±554.16	7468.83±901.72	P = 0.049
	Neighborhood Gray Tone Difference Matrix	Entropy	42398.74±2479.9	37595.91±2148.07	36320.06±969.14	P = 0.025
	Laws Texture Energy Measures	LL	111776.83±8124.23	100160.81±2589.38	101742.17±3658.35	P = 0.045
	Gray Level Difference Statistics	Variance	810.13±119.39	653.6±30.78	675.45±44.24	P = 0.011

Although the quantitative information from EB-OCT images that capture the features of malignant pulmonary nodules has been revealed, it is difficult for physicians to distinguish the normal and malignant lesions based on these features [9, 48] (also see **Fig 3B, 3D, 3F and 3I**). Automated image analysis methods by using artificial intelligence algorithm can provide a robust and accurate diagnosis independent of visual interpretation limitations. In order to involve complete information of one given patient, the training set and the testing set used in the current machine learning algorithm were classified according to different patient groups. One advantage of this setting is that the result deviation due to sampling bias could be avoided as much as possible since there was no intersection of OCT images between the training set and the testing set. All the quantitative features extracted from the EB-OCT images were used for classification of normal and malignant pulmonary nodules in RF algorithm. The average sensitivity, specificity, and accuracy were found to be 90.41%, 77.87% and 83.51%, respectively, for the testing datasets, which significantly outperforms the traditional clinical diagnosis of malignant pulmonary nodules with R-EBUS guided biopsy [38].

The current preliminary study has several limitations. Firstly, the training and testing results were based on a limited sample pool, and more data needs to be acquired for further validation. Secondly, the performance of the classification based on image features may be enhanced by further image processing, such as background or baseline corrections, updating the feature weights or filtering the noise attributes. In addition, the classification performance may be improved by employing a more robust learning algorithm, e.g., using deep convolutional neural networks, which does not need a manual or handcraft extraction of features [13]. Thirdly, the patients whose peripheral pulmonary nodules were less than 1 cm were excluded in this study, since they cannot be biopsied via bronchoscopy. However, these patients with small pulmonary nodules could benefit the most from the early identification of malignant lesions. For example, the surgeon would suggest surgical resection if the malignant rate of this kind of nodules is diagnosed to be high according to quantitative analysis of EB-OCT images associated with automated assessment by a well-trained classifier. Fourthly, the diameter of the EB-OCT catheter in this study was 1.7 mm. Due to the limitation of the physical properties of the catheter, it was difficult for the catheter to reach the lung lesions with large angles through the working channel of bronchoscope, e.g., the nodules located in the superior segment (S6). Therefore, only 2 cases of patients with superior segmental lesions were included in this study. Further investigation is necessary to recruit more patients to perform randomized controlled study, to validate the results and findings of this work with more clinically proven lung nodules.

**In conclusion**, quantitative features were extracted from 1703 EB-OCT images of pulmonary nodules of 31 patients, and the significant differences of image features between normal and malignant lesions were demonstrated. Using a RF classifier model, a sensitivity of 90.41%, specificity of 77.87% and accuracy of 83.51% was achieved to automated distinguish the normal and malignant pulmonary nodules. The promising results indicate that the EB-OCT combined with the machine leaning algorithm can potentially be a useful diagnostic tool for low cost identification of malignant pulmonary nodules. With more image data and addition of pathological information will make the system more robust and support in clinician decisions. We envision that our proposed method in future will assist specialists with early diagnosis, risk stratification, and prognosis of lung cancer patients.

## Supporting information

S1 Checklist STROBE statement—checklist of items that should be included in reports of observational studies. (DOCX)

S1 Fig An OCT image of healthy trachea. (DOCX)

S1 Video Video from radial endobronchial ultrasound. (MP4)

S2 Video Video from OCT. (AVI)

S1 Text Detailed definition and formulas of these image features. (DOCX)
