# Supplementary material for: Optical coherence tomography for identification of malignant pulmonary nodules based on random forest machine learning algorithm
Source: PLoS One. 2021 Dec 31;16(12):e0260600. doi: 10.1371/journal.pone.0260600 (PMC8719667; doi:10.1371/journal.pone.0260600)
Supplement: S1 Checklist — (DOCX) [file pone.0260600.s001.docx]

STROBE Statement—checklist of items that should be included in reports of observational studies

|  | Item No. | Recommendation | Page  No. | Relevant text from manuscript |
| --- | --- | --- | --- | --- |
| **Title and abstract** | 1 | (*a*) Indicate the study’s design with a commonly used term in the title or the abstract | 1 | Malignant pulmonary nodules |
|  |  | (*b*) Provide in the abstract an informative and balanced summary of what was done and what was found | 3 | A total of 31 patients with pulmonary nodules…. Attenuation coefficient and 29 image features were found to present different properties… |
| Introduction | | | |  |
| Background/rationale | 2 | Explain the scientific background and rationale for the investigation being reported | 3 | Pulmonary nodules are radiopaque densities seen in the lung parenchyma with a diameter of less than 3 cm… |
| Objectives | 3 | State specific objectives, including any prespecified hypotheses | 4 | The main objective of this study is to explore the feasibility of using automated image analysis system to classify pulmonary nodules in malignant and normal based on EB-OCT images… |
| Methods | | | |  |
| Study design | 4 | Present key elements of study design early in the paper | 4 | We investigated attenuation coefficient and up to 56 different image features extracted from A-line and B-scan of EB-OCT images. .. |
| Setting | 5 | Describe the setting, locations, and relevant dates, including periods of recruitment, exposure, follow-up, and data collection | 5 | This study group comprised 31 patients with solitary pulmonary nodule (SPN)… |
| Participants | 6 | (*a*) *Cohort study*—Give the eligibility criteria, and the sources and methods of selection of participants. Describe methods of follow-up  *Case-control study*—Give the eligibility criteria, and the sources and methods of case ascertainment and control selection. Give the rationale for the choice of cases and controls  *Cross-sectional study*—Give the eligibility criteria, and the sources and methods of selection of participants | 5 | his study group comprised 31 patients with solitary pulmonary nodule (SPN) who underwent EB-OCT and corresponding pathological examinations… |
|  |  | (*b*) *Cohort study*—For matched studies, give matching criteria and number of exposed and unexposed  *Case-control study*—For matched studies, give matching criteria and the number of controls per case | 5 | his study group comprised 31 patients with solitary pulmonary nodule (SPN) who underwent EB-OCT and corresponding pathological examinations… |
| Variables | 7 | Clearly define all outcomes, exposures, predictors, potential confounders, and effect modifiers. Give diagnostic criteria, if applicable | 7 | All patients received chest CT screening and chest CT thin-layer reconstruction to obtain the 0.65mm DICOM format CT data… |
| Data sources/ measurement | 8* | For each variable of interest, give sources of data and details of methods of assessment (measurement). Describe comparability of assessment methods if there is more than one group | *9* | *The extracted features can be subdivided into two categories: (1) A-line-derived optical properties and (2) B-scan-derived image features….* |
| Bias | 9 | Describe any efforts to address potential sources of bias | 9 | All the features were extracted from region of interest (ROI) with high signal-to-noise ratio (SNR) |
| Study size | 10 | Explain how the study size was arrived at | 2 | …up to 56 different image features were extracted from A-line and B-scan of 1703 EB-OCT images. |

Continued on next page

| Quantitative variables | 11 | Explain how quantitative variables were handled in the analyses. If applicable, describe which groupings were chosen and why | 9 | The extracted features can be subdivided into two categories: (1) A-line-derived optical properties and (2) B-scan-derived image features… |
| --- | --- | --- | --- | --- |
| Statistical methods | 12 | (*a*) Describe all statistical methods, including those used to control for confounding | 11 | The statistical software was SPSS 20.0 (SPSS Inc., Chicago, IL, USA) |
|  |  | (*b*) Describe any methods used to examine subgroups and interactions | 11 | The student’s t-test was used to investigate the differences between normal and malignant pulmonary nodules regarding to the attenuation coefficient and image features. A significance level of 0.05 was considered to be statistically significant. |
|  |  | (*c*) Explain how missing data were addressed | 19 | …since they cannot be biopsied via bronchoscopy |
|  |  | (*d*) *Cohort study*—If applicable, explain how loss to follow-up was addressed  *Case-control study*—If applicable, explain how matching of cases and controls was addressed  *Cross-sectional study*—If applicable, describe analytical methods taking account of sampling strategy | 19 | the patients whose peripheral pulmonary nodules were less than 1 cm were excluded in this study… |
|  |  | (*e*) Describe any sensitivity analyses | 11 | A significance level of 0.05 was considered to be statistically significant. |
| Results | | | | |
| Participants | 13* | (a) Report numbers of individuals at each stage of study—eg numbers potentially eligible, examined for eligibility, confirmed eligible, included in the study, completing follow-up, and analysed | 13 | The 31 patients selected in this study… |
|  |  | (b) Give reasons for non-participation at each stage | 19 | …since they cannot be biopsied via bronchoscopy |
|  |  | (c) Consider use of a flow diagram | N/A | N/A |
| Descriptive data | 14* | (a) Give characteristics of study participants (eg demographic, clinical, social) and information on exposures and potential confounders | 6 | Table 1 |
|  |  | (b) Indicate number of participants with missing data for each variable of interest | N/A | N/A |
|  |  | (c) *Cohort study*—Summarise follow-up time (eg, average and total amount) | 9 | a follow-up of every 3 months was conducted till the lesion was completely absorbed and confirmed to be normal |
| Outcome data | 15* | *Cohort study*—Report numbers of outcome events or summary measures over time | 6 | Table 1 |
|  |  | *Case-control study—*Report numbers in each exposure category, or summary measures of exposure |  |  |
|  |  | *Cross-sectional study—*Report numbers of outcome events or summary measures |  |  |
| Main results | 16 | (*a*) Give unadjusted estimates and, if applicable, confounder-adjusted estimates and their precision (eg, 95% confidence interval). Make clear which confounders were adjusted for and why they were included | 13 | Table 2 |
|  |  | (*b*) Report category boundaries when continuous variables were categorized | N/A | N/A |
|  |  | (*c*) If relevant, consider translating estimates of relative risk into absolute risk for a meaningful time period | 15 | As shown in Table 2, the attenuation coefficient of normal tissue is found to be higher than malignant one (0.055±0.018 vs. 0.050±0.019, p=0.044)… |

Continued on next page

| Other analyses | 17 | Report other analyses done—eg analyses of subgroups and interactions, and sensitivity analyses | 11 | All aforementioned features with significant p-values were used as predict variables in the classifier and RF model classified it into two classes… |
| --- | --- | --- | --- | --- |
| Discussion | | | | |
| Key results | 18 | Summarise key results with reference to study objectives | 18 | Using a RF classifier model, a sensitivity of 90.41%, specificity of 77.87% and accuracy of 83.51% was achieved to automated distinguish the normal and malignant pulmonary nodules |
| Limitations | 19 | Discuss limitations of the study, taking into account sources of potential bias or imprecision. Discuss both direction and magnitude of any potential bias | 18 | The current preliminary study has several limitations… |
| Interpretation | 20 | Give a cautious overall interpretation of results considering objectives, limitations, multiplicity of analyses, results from similar studies, and other relevant evidence | 18 | The average sensitivity, specificity, and accuracy were found to be 90.41%, 77.87% and 83.51%, respectively… |
| Generalisability | 21 | Discuss the generalisability (external validity) of the study results | 19 | The promising results indicate that  … |
| Other information | |  | | |
| Funding | 22 | Give the source of funding and the role of the funders for the present study and, if applicable, for the original study on which the present article is based | 1 | Fund for this project… |

*Give information separately for cases and controls in case-control studies and, if applicable, for exposed and unexposed groups in cohort and cross-sectional studies.

**Note:** An Explanation and Elaboration article discusses each checklist item and gives methodological background and published examples of transparent reporting. The STROBE checklist is best used in conjunction with this article (freely available on the Web sites of PLoS Medicine at http://www.plosmedicine.org/, Annals of Internal Medicine at http://www.annals.org/, and Epidemiology at http://www.epidem.com/). Information on the STROBE Initiative is available at www.strobe-statement.org.
