# Supplementary material for: Optical coherence tomography for identification of malignant pulmonary nodules based on random forest machine learning algorithm
Source: PLoS One. 2021 Dec 31;16(12):e0260600. doi: 10.1371/journal.pone.0260600 (PMC8719667; doi:10.1371/journal.pone.0260600)
Supplement: S1 Fig — (DOCX) [file pone.0260600.s002.docx]

**Supplementary material S1 Figure**


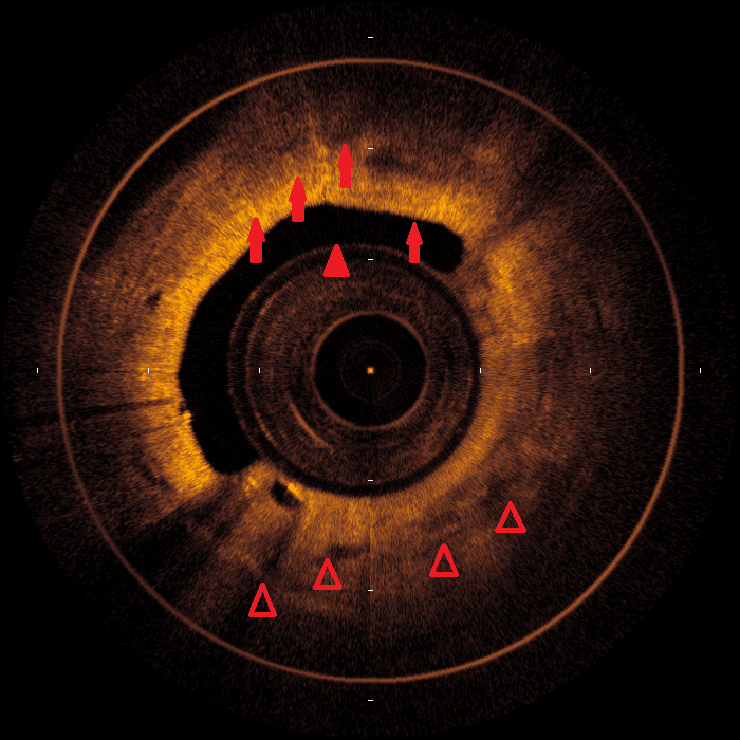


An OCT image of a normal distal airway. The size of the lumen is approximately 2 mm. The red triangles point to the air inside the airway. The image shows a clear hierarchy of airway structures, including the mucosal layer, the submucosal layer, and the outer layer (red arrows). The other side of the probe is pressed against the airway wall to show a clear image of the alveoli (red hollow triangle).
