# Supplementary material for: Optical coherence tomography for identification of malignant pulmonary nodules based on random forest machine learning algorithm
Source: PLoS One. 2021 Dec 31;16(12):e0260600. doi: 10.1371/journal.pone.0260600 (PMC8719667; doi:10.1371/journal.pone.0260600)
Supplement: S1 Text — (DOCX) [file pone.0260600.s005.docx]

**Optical Coherence Tomography for Identification of Malignant Pulmonary Nodules based on Random Forest Machine Learning Algorithm**

**Ming Ding^*^** ^1^ **Ph.D., Shi-yu Pan^*^**^2^ **M.S.,** **Jing Huang**^1^ **M.D., Cheng Yuan**^1^ **M.D., Qiang Zhang ^1^Ph.D., Xiao-li Zhu** ^1^ **Ph.D., Yan Cai^2^, Ph.D.**

1 Department of Respiratory Medicine, Southeast University Zhongda Hospital, Nanjing, Jiangsu, China.

2 School of Biological Sciences and Medical Engineering, Southeast University, Nanjing, Jiangsu, China.

**Appendix**

1. Statistical feature (SF)

The Statistical features^[1]^ were extracted quantitatively from the analysis of the normalized histogram of images. If N represents the total number of an image, and $X_{i}$ is the $i^{th}$ pixel gray level, then the four features can be calculated from these expressions:

Mean $(\mu) =\frac{1}{N}\sum_{i=1}^{N} X_{i}$

Standard deviation $(\sigma) =\sqrt{\frac{1}{N}\sum_{i=1}^{N} \left( X_{i}-\mu\right)^{2}}$

Skewness $=\frac{1}{N}\sum_{i=1}^{N} \left[ \frac{(X_{i}-\mu)}{\sigma} \right]^{3}$

Kurtosis $=\frac{1}{N}\sum_{i=1}^{N} \left[ \frac{(X_{i}-\mu)}{\sigma} \right]^{4}-3$

Mean is the average gray pixel intensity. The standard deviation of the histogram distribution measures can be measured from its mean value. Skewness and kurtosis indicate the flatness and asymmetry of the histogram.

1. Fractal dimension analysis (FDA)

Mandelbrot developed the fractional Brownian motion model to the roughness of natural surfaces^[2,3]^. The Hurst coefficient $H^{(w)}$ was computed ^[4]^ for image resolutions w=1,2,3,4. Fractal dimension $D_{f}$ can be computed from the relationship^[3]^

$$D_{f}=3-H$$

A small value of $D_{f}$ means a smooth surface, and large $D_{f}$ a rough surface.

1. Fourier power spectrum (FPS)

Different from other methods, this method extracts the texture features in the frequency domain, which also contains textural information^[5-7]^. To use the Fourier power spectrum, one must first perform discrete Fourier transform:

$$F\left( u,v \right)=\sum_{x=0}^{M-1} \sum_{y=0}^{N-1} f\left( x,y \right)exp\left[ -j2\pi\left( \frac{ux}{M}+\frac{vy}{N} \right) \right]$$

Where $f\left( x,y \right)$ represents an image of size M×N, and $F\left( u,v \right)$ stands for the Fourier transform of the image. Since $F\left( u,v \right)$ is an imaginary number, we use the power spectrum as the texture feature of the image:

$$E\left( u,v \right)=\left| F\left( u,v \right) \right|^{2}$$

Radial sum and angular sum are commonly used for FPS features, which can be calculated respectively from equations below:

$$\emptyset_{r1,r2}\equiv\sum_{{r1}^{2}\leq u^{2}+v^{2}<{r2}^{2}} \left| F\left( u,v \right) \right|^{2}$$

$$\emptyset_{\theta1,\theta2}\equiv\sum_{\theta1\leq\tan^{-1} \left( \frac{v}{u} \right)<\theta2} \left| F\left( u,v \right) \right|^{2}$$

Where 0 < u < M-1, 0 < v < N-1 for a given M×N image.

1. Spatial gray level dependence matrices (SGLDM)

The spatial gray level dependence matrices are based on the estimation of the second-order joint conditional probability density functions $P\left( i,j;d,\theta\right)$ is the probability of going from gray-level $i$ to gray-level $j$, given that the sample spacing is $d$ and the direction is specified by the angle $\theta$. Formally, for angles quantized to 45° intervals the unnormalized frequencies are defined by^[5,8]^

where # denotes the number of elements in the set. $L_{x}$ and $L_{y}$, are the horizontal and vertical spatial domains, $I\left( x,y \right)$ is the image intensity at point $\left( x,y \right)$.

Haralick proposed 14 measures that can be employed to extract texture information, and 13 of the measures are applied in our experiment. They are as follow:

1. Angular Second Moment:

$$f_{1}=\sum_{i} \sum_{j} \left\{ p(j,j) \right\}^{2}$$

2. Contrast:

$$f_{2}=\sum_{n=0}^{N_{g-1}} n^{2}\left\{ \sum_{i=1}^{N_{g}} \sum_{j=1}^{N_{g}} p(j,j) \right\} \left| i-j \right|=n$$

3. Correlation:

$$f_{3}=\frac{\sum_{i} \sum_{j} \left( ij \right)p\left( i,j \right)-\mu_{x}\mu_{y}}{\sigma_{x}\sigma_{y}}$$

4. Sum of Squares: Variance

$$f_{4}=\sum_{i} \sum_{j} \left( i-\mu\right)^{2}p(i,j)$$

5. Inverse Difference Moment:

$$f_{5}=\sum_{i} \sum_{j} \frac{1}{1+{(i-j)}^{2}} p(i,j)$$

6. Sum Average:

$$f_{6}=\sum_{i=2}^{2N_{g}} ip_{x+y}(i)$$

7. Sum Variance:

$$f_{7}=\sum_{i=2}^{2N_{g}} {(i-f_{8})}^{2}p_{x+y}(i)$$

8. Sum Entropy:

$$f_{8}=-\sum_{i=2}^{2N_{g}} p_{x+y}\left( i \right)\log\{p_{x+y}(i)\}$$

9. Entropy:

$$f_{9}=-\sum_{i} \sum_{j} p(i,j)log( p(i,j))$$

10. Difference Variance:

$$f_{10}=variance of p_{x-y}$$

11. Difference Entropy:

$$f_{11}=-\sum_{i=0}^{N_{g-1}} p_{x-y}\left( i \right)\log\{p_{x-y}(i)\}$$

12. Information Measures of Correlation:

$$f_{12}=\frac{HXY-HXY1}{max\{HX,HY\}}$$

$$f_{13}={(1-\exp\left[ -2.0\left( HXY2-HXY \right) \right])}^{1/2}$$

$$HXY=-\sum_{i} \sum_{j} p(i,j)log( p(i,j))$$

Where HX and HY are entropies of $p_{x}$ and $p_{y}$, and

$$HXY1=-\sum_{i} \sum_{j} p\left( i,j \right)\log\{p_{x}(i)p_{y}(j)\}$$

$$HXY2=-\sum_{i} \sum_{j} p_{x}(i)p_{y}(j)\log\{p_{x}(i)p_{y}(j)\}$$

Each measure is evaluated for $d=1$ and $\theta=0^{\circ},45^{\circ},90^{\circ},135^{\circ}$. The features are obtained from the mean and range of the measure values.

1. Gray level difference statistics (GLDS)

To describe the gray level difference statistics, let $I(x,y)$ be the image intensity function. For any given displacement $\delta\equiv\left( \Delta x,\Delta y \right)$, let $I_{\delta}\left( x,y \right)\equiv\left| I\left( x,y \right)-I(x+\Delta x,y+\Delta y) \right|$ be gray difference. $I_{\delta}\left( x,y \right)$ of each pixel is calculated by moving $\left( x,y \right)$ throughout the image.^[8]^ If there are $m$ gray levels, this has the form of an $m$-dimensional vector whose $i$th component is the number that $I_{\delta}\left( x,y \right)$ will have value $i$. It is easy to compute the probability density $p_{\delta}$ of $I_{\delta}\left( x,y \right)$ by counting the number of times each value of $I_{\delta}\left( x,y \right)$ occurs where $\Delta x$ and $\Delta y$ are integers.

The features used to classify in this study are as follow:

$$Homogeneity\equiv\sum_{i} (\frac{p_{\delta}\left( i \right)}{i^{2}+1})$$

$$Contrast\equiv\sum_{i} i^{2}p_{\delta}(i)$$

$$Energy\equiv\sum_{i} {p_{\delta}(i)}^{2}$$

$$Entropy\equiv-\sum_{i} p_{\delta}(i)\log p_{\delta}(i)$$

$$Mean\equiv\frac{1}{m}\sum_{i} {ip}_{\delta}(i)$$

The measure are estimated for $\delta\equiv\left( 1,0 \right), \left( 1,1 \right), \left( -1,1 \right),$ and $\left( 0,1 \right)$ and take the sample mean.

1. Neighborhood gray tone difference matrix (NGTDM)

The NGTDM defines features related to human perception of a texture^[9]^. We assume $s(i)$ is a column matrix formed by summing the absolute value of the pixel minus the average of pixels in its neighborhood. In this experiment, the neighborhood was defined as $d=1$ pixels and five features were calculated:

$$Coarseness=\left[ \sum_{i=1}^{L} p_{i}s(i) \right]^{-1}$$

$$Contrast=\frac{1}{N_{g}\left( N_{g}-1 \right)}\sum_{i=1}^{L} \sum_{j=1}^{L} p_{i}p_{j}{(i-j)}^{2}\cdot\frac{1}{n^{2}}\sum_{i=1}^{L} s(i)$$

$$Busyness=\sum_{i=1}^{L} p_{i}s(i)/\sum_{i=1}^{L} \sum_{j=1}^{L} {(ip}_{i}{-jp}_{j})$$

$$Complexity=\sum_{i=1}^{L} \sum_{j=1}^{L} \{(\left| i-j \right|)/(n^{2}\left( p_{i}+p_{j} \right))\}\{p_{i}s\left( i \right)+p_{j}s(j)\}$$

$$Strength=\sum_{i=1}^{L} \sum_{j=1}^{L} (p_{i}+p_{j}){(i-j)}^{2}/\sum_{i=1}^{L} s(i)$$

where $N_{g}$ is the total number of gray levels in the image. If the size of an image is $N\times N$, $n=N-2d$.

1. Statistical feature matrix (SFM)

SFM is a method that directly evaluates the statistical features for several intersample spacing distances from the image^[10]^. The features used in our study are as follow:

1. Coarseness

$$F_{crs}=c/m_{crs}$$

Where c is a normalizing factor and

$$m_{crs}=\sum_{(i,j)\in N_{r}} DSS(i,j)/n$$

$$DSS\left( i,j \right)= E\left\{ |I\left( x,y \right)-I\left( x+\Delta x,y+\Delta y \right)| \right\}$$

Where $E\left\{ \cdot\right\}$ denotes the expectation operation.

1. Contrast

$$CON(\delta)=E\left\{ \left[ I\left( x,y \right)-I(x+\Delta x,y+\Delta y) \right]^{2} \right\}$$

Where $E\left\{ \cdot\right\}$ denotes the expectation operation.

1. Periodicity

$$F_{per}=\frac{\bar{M_{dss}}-M_{dss}(valley)}{\bar{M_{dss}}}$$

Where $\bar{M_{dss}}$ is the mean of all elements of $M_{dss}$, and $M_{dss}(valley)$ is the deepest valley in the matrix.

1. Roughness

$$F_{rgh}=(D_{f}^{\left( h \right)}+D_{f}^{\left( v \right)})/2$$

Where $D_{f}^{\left( h \right)}$ and $D_{f}^{\left( v \right)}$ are the estimated fractal dimensions in horizontal and vertical directions.

1. Laws’ texture energy measures (LTEM)

Laws’ texture energy measures^[4,11-13]^ are derived from three simple vector of length 3 which are defined as kernel, $L3\equiv(1,2,1)$, $E3\equiv\left( -1,0,1 \right), and S3\equiv(-1,2,-1)$. If these vectors are convolved with themselves, we obtain other three vectors of length 5, $L5\equiv(1,4,6,4,1)$,$E5\equiv\left( -1,-2,0,2,1 \right)$, $S5\equiv(-1,0,2,0,-1)$. If these vectors are convolved with themselves, we obtain three vectors of length 5, $L7\equiv(1,6,15,20,15,6,1)$,$E7\equiv\left( -1,-4,-5,0,5,4,1 \right)$, $S7\equiv(-1,-2,1,4,1,-2-1)$ where L7 performs local averaging, S7 and E7 are spot and edge detectors respectively. If we multiply the column vectors of length 7 by row vectors of the same length, we obtain Laws’ $7\times7$ masks. To use these masks to describe texture in an image, we convolve them with the image and use statistics(e.g. energy) of the results as texture properties. The features we used in our experiments are LL-texture energy from LL kernel, EE-texture energy from EE-kernel, SS-texture energy from SS-kernel, LE-average texture energy from LE and EL kernels, ES-average texture energy from ES and SE kernels, and LS-average texture energy from LS and SL kernels.

1 Press, W. H., Teukolsky, S. A., Vetterling, W. T. & Flannery, B. P. *Numerical recipes 3rd edition: The art of scientific computing*. (Cambridge university press, 2007).

2 Chakerian, D. (JSTOR, 1984).

3 Shanmugavadivu, P. & Sivakumar, V. Fractal dimension based texture analysis of digital images. *Procedia Engineering* **38**, 2981-2986 (2012).

4 Wu, C.-M., Chen, Y.-C. & Hsieh, K.-S. Texture features for classification of ultrasonic liver images. *IEEE Transactions on medical imaging* **11**, 141-152 (1992).

5 Haralick, R. M., Shanmugam, K. & Dinstein, I. H. Textural features for image classification. *IEEE Transactions on systems, man, and cybernetics*, 610-621 (1973).

6 Kruger, R. P., Thompson, W. B. & Turner, A. F. Computer diagnosis of pneumoconiosis. *IEEE Transactions on Systems, Man, and Cybernetics*, 40-49 (1974).

7 Lendaris, G. G. & Stanley, G. L. Diffraction-pattern sampling for automatic pattern recognition. *Proceedings of the IEEE* **58**, 198-216 (1970).

8 Weszka, J. S., Dyer, C. R. & Rosenfeld, A. A comparative study of texture measures for terrain classification. *IEEE transactions on Systems, Man, and Cybernetics*, 269-285 (1976).

9 Amadasun, M. & King, R. Textural features corresponding to textural properties. *IEEE Transactions on systems, man, and Cybernetics* **19**, 1264-1274 (1989).

10 Wu, C.-M. & Chen, Y.-C. Statistical feature matrix for texture analysis. *CVGIP: Graphical Models and Image Processing* **54**, 407-419 (1992).

11 Laws, K. I. in *Proc. Image understanding workshop.* 47-51.

12 Laws, K. I. in *Image processing for missile guidance.* 376-381 (International Society for Optics and Photonics).

13 Pietikäinen, M., Rosenfeld, A. & Davis, L. S. Experiments with texture classification using averages of local pattern matches. *IEEE transactions on systems, man, and cybernetics*, 421-426 (1983).
